# Advances in CO_2_ utilization employing anisotropic nanomaterials as catalysts: a review

**DOI:** 10.3389/fchem.2023.1175132

**Published:** 2023-05-25

**Authors:** Vishal Kandathil, Narayanapillai Manoj

**Affiliations:** Department of Applied Chemistry, Inter University Center for Nanomaterials and Devices, Cochin University of Science and Technology, Kochi, India

**Keywords:** anisotropic nanomaterials, CO_2_ utilization, sustainability, green chemistry, catalysis

## Abstract

Anisotropic nanomaterials are materials with structures and properties that vary depending on the direction in which they are measured. Unlike isotropic materials, which exhibit uniform physical properties in all directions, anisotropic materials have different mechanical, electrical, thermal, and optical properties in different directions. Examples of anisotropic nanomaterials include nanocubes, nanowires, nanorods, nanoprisms, nanostars, and so on. These materials have unique properties that make them useful in a variety of applications, such as electronics, energy storage, catalysis, and biomedical engineering. One of the key advantages of anisotropic nanomaterials is their high aspect ratio, which refers to the ratio of their length to their width, which can enhance their mechanical and electrical properties, making them suitable for use in nanocomposites and other nanoscale applications. However, the anisotropic nature of these materials also presents challenges in their synthesis and processing. For example, it can be difficult to align the nanostructures in a specific direction to impart modulation of a specific property. Despite these challenges, research into anisotropic nanomaterials continues to grow, and scientists are working to develop new synthesis methods and processing techniques to unlock their full potential. Utilization of carbon dioxide (CO_2_) as a renewable and sustainable source of carbon has been a topic of increasing interest due to its impact on reducing the level of greenhouse gas emissions. Anisotropic nanomaterials have been used to improve the efficiency of CO_2_ conversion into useful chemicals and fuels using a variety of processes such as photocatalysis, electrocatalysis, and thermocatalysis. More study is required to improve the usage of anisotropic nanomaterials for CO_2_ consumption and to scale up these technologies for industrial use. The unique properties of anisotropic nanomaterials, such as their high surface area, tunable morphology, and high activity, make them promising catalysts for CO_2_ utilization. This review article discusses briefly about various approaches towards the synthesis of anisotropic nanomaterials and their applications in CO_2_ utilization. The article also highlights the challenges and opportunities in this field and the future direction of research.

## 1 Introduction

When applied to the field of materials chemistry, the notion of sustainability aligns with many of the tenets of “green chemistry” (Anastas and Eghbali, 2010). These principles include the utilization of precursors that are lower in toxicity in the preparation of nanomaterials, which includes utilizing water, in circumstances when it is possible, as a solvent; the utilization of the fewest number of reagents and the fewest number of synthetic steps wherever feasible; the minimization of by-products and waste; and the utilization of a reaction temperature that is relatively close to that of the room temperature (Anastas and Eghbali, 2010; Chen et al., 2020). A major way to accomplish the goals of sustainable (green) chemistry is by using catalysis which can minimize the use of resources and energy (Anastas et al., 2000; Centi and Perathoner, 2003; Horvath and Anastas, 2007). The Environmental Protection Agency (EPA) established the discipline of “green chemistry” in response to the growing demand for more environmentally responsible processes in the chemical industry (Anastas and Williamson, 1996). The goal of green chemistry is to minimize or eliminate the use of toxic and hazardous substances and to cut down on or eliminate waste generated from chemical reactions without sacrificing the effectiveness of the processes. In this context, Paul Anastas and John C. Warner proposed a set of fundamental principles with the intention of elaborating the requirements of green chemistry and further guiding their implementation in chemical processes (Anastas and Warner, 1998). Nanoparticle synthesis has garnered a huge amount of interest in recent times due to the fact that it produces functional nanoparticles that have a wide range of applications in different areas, including medicine, catalysis, sensing, electronics, and photonics (Shipway et al., 2000; Fedlheim and Foss, 2001; Campelo et al., 2009; Liu and Tang, 2010; Stavis et al., 2018; Rider et al., 2022).

**FIGURE 1:**
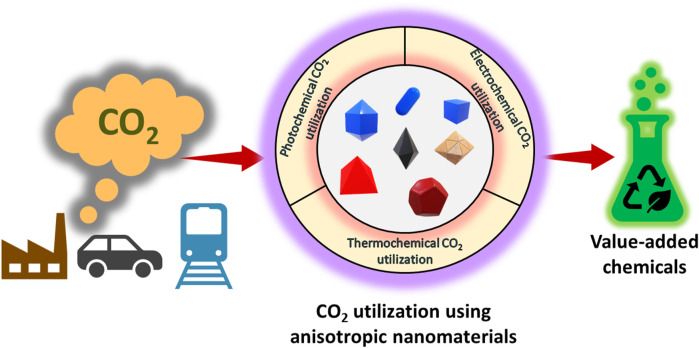
A graphic representation of anisotropic nanomaterials for CO_2_ utilization by different approaches.

Catalysts play a major role in various reactions and are widely employed for their efficient product output. Among the catalysts, heterogeneous catalysts are widely used in the industrial sector when compared with the homogeneous ones (Zaera, 2022). The efficiency of heterogeneous catalysts is comparatively lower when compared with their homogeneous counterparts, the reason being the agglomeration of active sites, less dispersion, and lower surface area of heterogeneous catalysts. But at the same time, the possibility of recovering the catalyst after the completion of the reaction and its recyclability are the key advantages of the heterogeneous catalysts (Astruc et al., 2005; Thomas et al., 2005). The introduction of nanocatalysts with a high surface-to-volume ratio has been a significant step toward improving the efficiency of heterogeneous catalysts, but efficiency and activity remain major concerns when considering them for longer cycles (Polshettiwar et al., 2011). One way to enhance the efficiency of nanocatalysts is by introducing anisotropy into their structures (Li and Shen, 2014). The shape and morphology of nanomaterials can play an important role in their properties. The improved efficiency of anisotropic nanomaterials may be ascribed to a break in symmetry, which allows for new physicochemical features (Li and Shen, 2014; Cuenya and Behafarid, 2015). Figure 1 shows a graphic representation of anisotropic nanomaterials for CO_2_ utilization by different approaches. The synthesis of nanomaterials with varied shape and morphology gained momentum after the invention of carbon nanotubes, which possess unique and astonishing physicochemical properties (Gupta et al., 2019; Swain et al., 2020). Hence, a lot of research is now being carried out to engineer the shape and morphology of nanomaterials.

One can foresee many practical uses for these green nanocatalysts. Global warming and its control are major concern for humanity and life on earth at large. To address this a viable strategy is to control one of the causative elements the level of CO_2_ in the atmosphere. Photosynthesis by plants is the major pathway in the fixation of CO_2_, however, this is not sufficient and newer methods of CO_2_ fixation need to be developed (Al-Ghussain, 2019). One such strategy is by utilizing the CO_2_ in the synthesis of other value-added chemicals and fuels using green nanocatalysts (Tsuji and Fujihara, 2012; Yaashikaa et al., 2019). Among the various strategies employed currently for the utilization of CO_2_, the catalytic reduction of CO_2_ is important (Modak et al., 2020). There are many approaches available for the efficient CO_2_ reduction reaction, including photochemical, thermochemical, and electrochemical pathways with very rapid reaction rates and energy efficiency along with the production of value-added products (Olajire, 2013; Zheng et al., 2017; Mustafa et al., 2020). Since mid-2010, academic researchers have steadily expanded their focus on enhancing efficiency, stability, selectivity, and environmental benignness for effective CO_2_ utilization while lowering costs (Aresta et al., 2013). This also resulted in an exponential rise in the number of research publications addressing the CO_2_ reduction reaction (Aresta et al., 2013; Cao et al., 2016; Valluri et al., 2022). Since, there has recently been a boom in research in the field of anisotropic nanomaterials and there exists a vast source of associated literature, a thorough evaluation of research activity is a difficult endeavor (Sajanlal et al., 2011; Li et al., 2014; Thorkelsson et al., 2015; Bhol et al., 2020; Zeng et al., 2020; Pearce et al., 2021). Only a few intriguing and important aspects of research in this area is the focal theme of this review article.

## 2 Anisotropic nanomaterials

Anisotropic nanomaterials acquire special attributes due to their increased surface-to-volume ratio and the spatial confinement of charges (electrons, phonons, and electric fields) surrounding the particles (Sajanlal et al., 2011). The nature of electron movement has a substantial influence on the physicochemical characteristics of these materials. Anisotropic nanomaterials may be given new and astounding characteristics when we limit the mobility of electrons through the construction of appropriate shapes for the materials (Thorkelsson et al., 2015). Free electrons have unquantized motion and may consequently absorb energy without restrictions. But when an electron is confined in an atom or molecule, its mobility is fairly limited, and quantization occurs. Whenever the region in which mobility is restricted is smaller, the confinement gets tighter, and the energy separation among the permissible energy levels gets wider (Thorkelsson et al., 2015). Since electrons in isotropic materials, like spheres (0-dimensional), are confined to a similar extent in all three dimensions, the characteristics of such materials are basically the same in any given direction. Also, isotropic nanomaterials tend to aggregate at random, making precise arrangements difficult and limiting their potential in numerous applications, such as nanoelectronics and photonics. In addition, as anisotropic properties such as directional conductivity or polarizability are not inherent, isotropic nanomaterials may lack specific functionalities required for certain applications. The properties of isotropic nanomaterials depend on size, shape, and composition, but their range of achievable properties is often limited when compared to anisotropic nanomaterials. However, to obtain anisotropic structures, the challenge is aligning constituent molecules in a certain orientation during synthesis, and a lack of it can limit their performance (Liu et al., 2008). Despite these obstacles, scientists are attempting to create innovative synthesis processes and methods so as to generate newer classes of anisotropic nanomaterials with distinct properties. In the future, it is anticipated that anisotropic nanomaterials will play a vital role in a vast array of technologies, from nanoelectronics to energy generation and storage, sensing, catalysis, and so on.

## 3 Synthesis of anisotropic nanomaterials

### 3.1 Template-assisted synthesis

**FIGURE 2:**
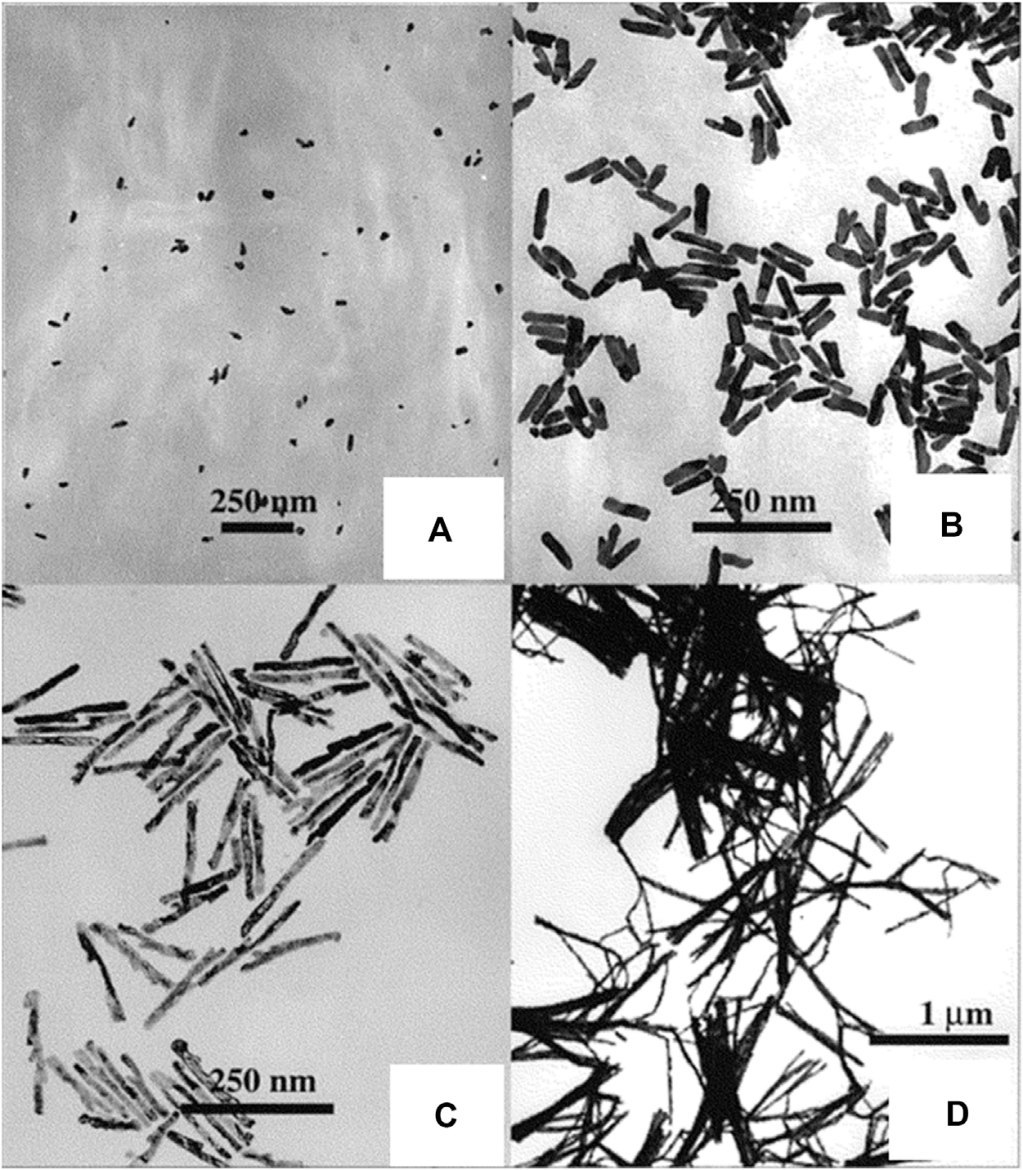
TEM micrographs of gold rods: **(A)** *L* = 40 nm, *d =* 22 nm; **(B)** *L =* 82 nm, *d =* 19 nm; **(C)** *L =* 189 nm, *d =* 15 nm; **(D)** *L* = 729 nm, *d* = 15 nm [Reprinted with permission from Ref. (Van der Zande et al., 2000), American Chemical Society].

The template-mediated approach has emerged as one of the most prominent ways for synthesizing 1D nanostructures that have uniform size and seem to have physical dimensions that can be controlled (Meng et al., 2009; Liang et al., 2010). The main advantages of this method over others are its ease of fabrication, lower cost, maximum output, and adaptability to a wide range of material compositions. In this approach, generally alumina or nanoporous polycarbonate is employed as the template, and the procedure depends on the electrochemical deposition of metals in the assembly of the template. To form a conductive layer for electrodeposition, a little amount of metal is first sputtered on the template. The template is then deposited with the metal, which is to be made into anisotropic nanomaterials, via an electrochemical process. Selective dissolution is then carried out to remove the Ag or Cu-based conductive film and the template, while a polymeric stabilizer is present. Lastly, using sonication, the prepared anisotropic nanomaterials are dispersed in a suitable solvent. Figure 2 shows the transmission electron microscopy (TEM) images of gold nanorods (NRs) prepared by the template-mediated synthesis by Van der Zande et al. (Van der Zande et al., 2000). Salem et al. electrodeposited nickel into nanoporous alumina templates to construct self-organized nickel nanowire arrays with tunable dimensions and examined how the annealing process affected the structural and magnetic characteristics of the nanowires (Salem and Mahdy, 2022). Potential applications for NiO nanowires include their use in supercapacitors, non-volatile memory, LEDs, and so on. A new polymer-templated synthesis technique for Ag/Au bimetallic nanoparticles (BNPs) was recently reported by Fahes et al. and was used in sensing applications (Fahes et al., 2022). Different structures of BNPs, such as heterostructures, eccentric core-shells, and physical mixtures of two metals, were synthesized by regulating the reaction kinetics. Because of their strong surface-enhanced Raman scattering (SERS) sensitivity, the resultant anisotropic BNPs were excellent for sensitive 4,4′-bipyridine sensing. The results indicate that this approach to surfactant-free synthesis is a potent tool for developing growth strategies based on silicon substrate platforms. The real-time radiolytic growth of Ag@Au core-shell nanostructures using *in situ* liquid-cell transmission electron microscopy via template-assisted synthesis was reported by Ahmad et al. (Ahmad et al., 2019). Herein, the growth process was controlled by using a capping agent and a coordinating complex in an organic solvent to slow down the reaction kinetics and shift the growth regime from galvanic replacement mode to direct synthesis mode. Classical simulations were used to complement the experimental results and provide further insight on the growth modes. This report focuses on the shape evolution and chemical ordering of the nanoalloys, highlighting the impact of extrinsic parameters such as additives, capping agents, and modulation of the surface energies of exposed crystal surfaces by the encapsulating solvent in template-mediated synthesis.

### 3.2 Seed-mediated synthesis

**FIGURE 3:**
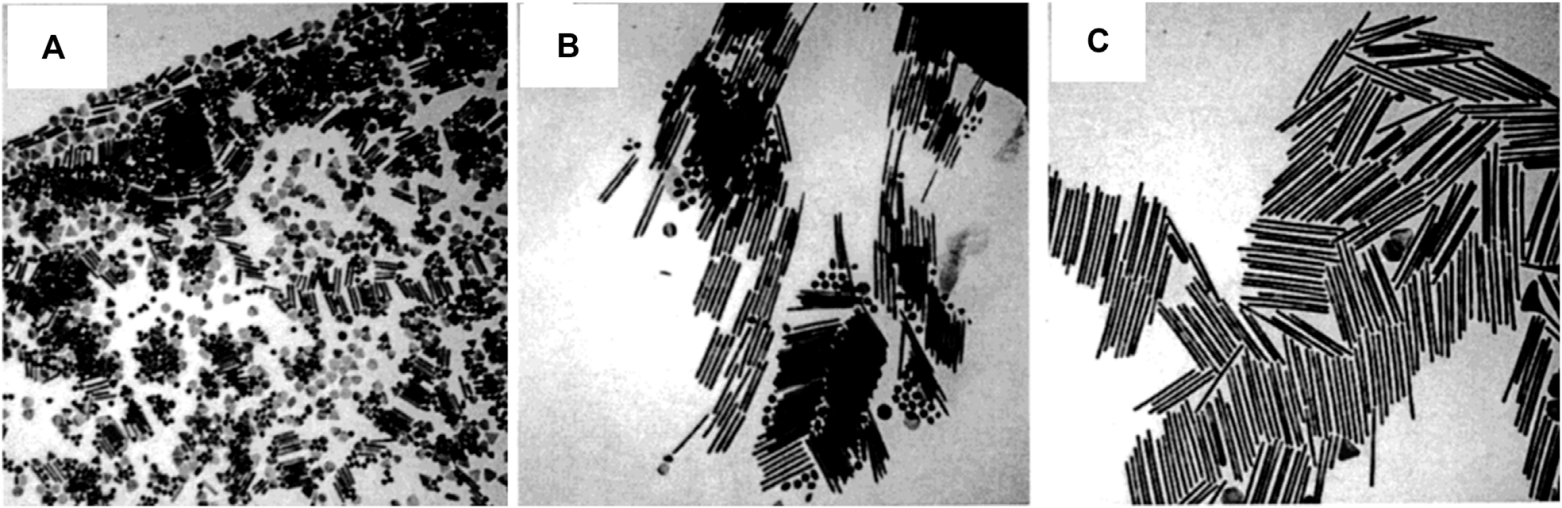
**(A)** TEM images of 4.6 aspect ratio gold nanorods, **(B)** shape-separated 13 aspect ratio gold nanorods, and **(C)** shape-separated 18 aspect ratio gold nanorods. The scale bar (100 nm) applies to all three images. [Reprinted with permission from Ref. (Jana et al., 2001), American Chemical Society].

Despite the fact that several ways have been employed for anisotropic nanostructure fabrication, the seed-mediated synthesis approach is an extensively used one that may generate a variety of nanostructures, including wires, triangles, rods, and so on (Jana et al., 2001; Fan et al., 2008). This approach involving a two-step procedure is an adapted version of Zsigmondy’s ‘nuclear’ method (Zsigmondy, 1917). The seed nanoparticles are synthesized first by a facile reduction reaction with stabilizing agents present, wherein the reducing agents like sodium borohydride will reduce the metal salt precursor. The second stage of this process involves the transformation of the seed nanoparticles into the required shape, which is achieved by the shaping agent, or surfactant, and a reducing agent present in the growth solution. During this process, the metal precursors will undergo reduction on the seed nanoparticle surface. The desired morphology is achieved by the surfactant molecules that form proper templates that aid in the development process. By altering the quantity of seed nanoparticles, it is feasible to alter the size of the nanoparticles to be made. The gold NRs synthesized by Jana et al. (Jana et al., 2001) are shown in Figure 3. The synthesis of hybrid nanoparticles consisting of bismutite nanodisks and gold nanoparticles with different morphologies, such as spheres, rods, and etched rods, was reported by Antony et al. (Antony et al., 2022). The authors used a modified seed-mediated growth method to control the shape of the gold nanoparticles before depositing them on the substrate. By fine-tuning the shape of the gold nanoparticles, the authors were able to enhance the light-harvesting capabilities of the hybrid nanoparticles. Qiao et al. came up with a stable seed-mediated synthesis method for making Au nanoplates with less thickness, a high morphological yield, and optical properties that can be controlled (Qiao et al., 2023). Here, authors illustrate how to make uniformly thin Au nanoplates by utilizing Au-Ag alloy nanoframes produced by galvanic replacement of Ag nanoplates with HAuCl_4_ and a sulfite (SO_3_^2-^) as seeds and ligands, respectively.

### 3.3 Biological synthesis

**FIGURE 4:**
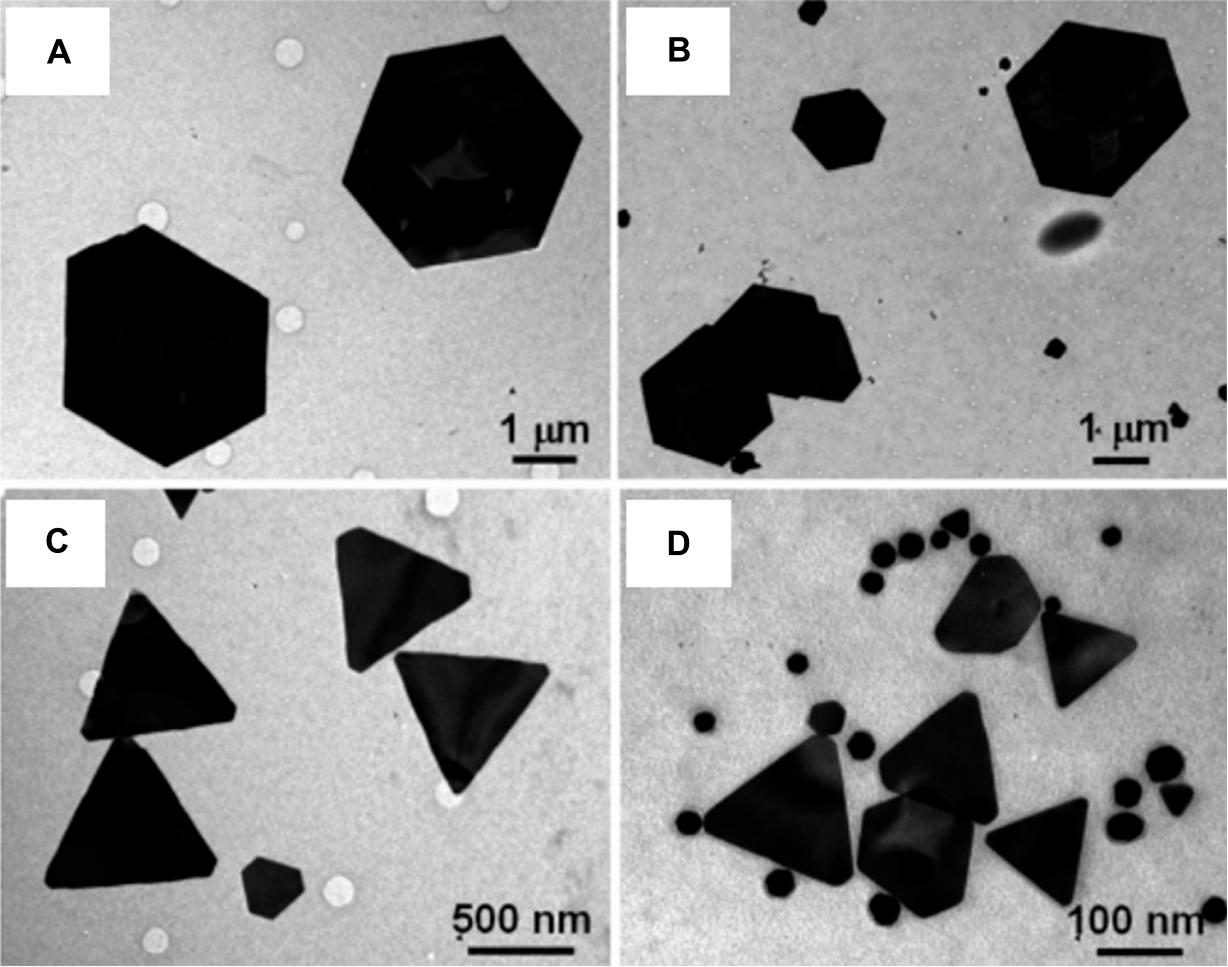
Representative TEM images of gold nanoparticles synthesized by the reduction of 5 mL of 10–3 M aqueous HAuCl_4_ solution with **(A)** 0.2, **(B)** 0.3, **(C)** 0.5, and **(D)** 1.0 mL of lemon grass extract. [Reprinted with permission from Ref. (Shankar et al., 2005), American Chemical Society].

Inorganic nanomaterials like magnetite, calcite, or amorphous silica can be used to make functional superstructures by biological synthesis (Yu, 2007). The synthesis of nanomaterials with controlled morphology in biological systems has been accomplished either via growth in confined surroundings, like membrane vesicles, or via functional molecules, like polypeptides, that selectively bind to the inorganic surfaces of crystallographic planes. For instance, *Pseudomonas* stutzeri AG259, a bacterial strain that was isolated from a silver mine, has been used to biologically synthesize silver nanoparticles with hexagonal and equilateral triangle morphologies (Klaus et al., 1999). By using the repeat sequences of specific polypeptide proteins released by the *Escherichia coli* bacterium, flat and triangular gold nanocrystals were prepared (Brown et al., 2000). It was discovered by Shiv Shankar et al. that the reducing sugars (aldoses) in the extract of lemongrass could reduce the gold precursor to nanoprisms (Figure 4) (Shankar et al., 2005). Also, the size of the nanoprisms may be altered by simply adjusting the quantity of lemongrass extract present in the reaction medium, which makes it feasible to readily control the longitudinal surface plasmon resonance (SPR) band in the near infrared (NIR) region.

### 3.4 Polyol synthesis

**FIGURE 5:**
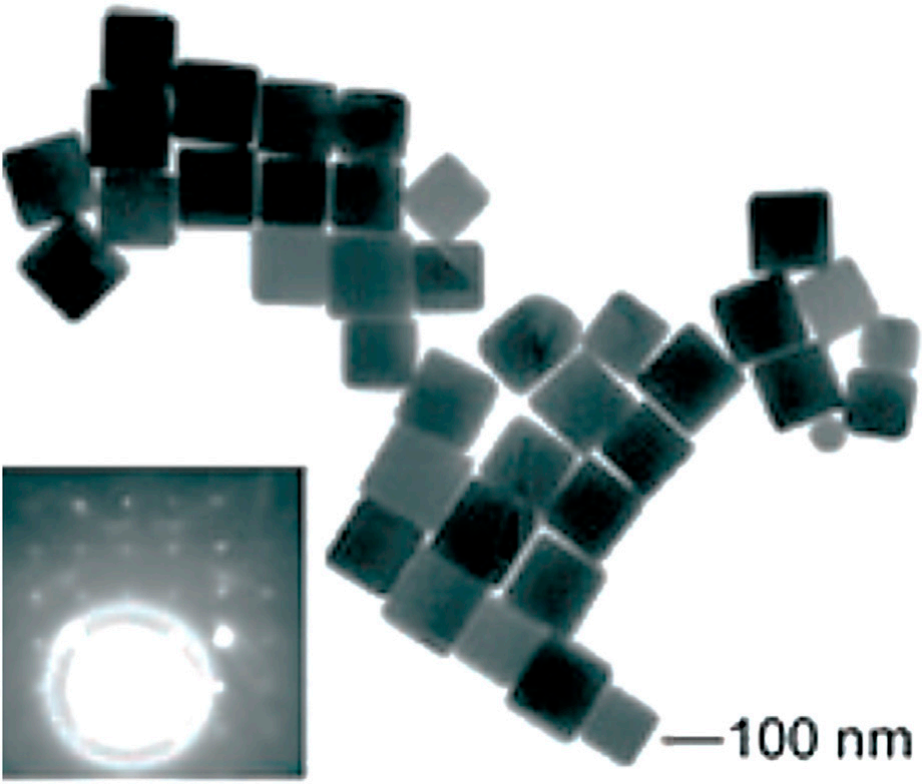
TEM image of the silver nanocubes. The inset shows an electron diffraction pattern recorded by directing the electron beam perpendicular to the (100) facet of a silver nanocube. [Reprinted with permission from Ref. (Im et al., 2005), John Wiley & Sons, Inc.].

Fiévet et al. (Fievet et al., 1989) and Viau et al. (Viau et al., 1996) developed the straightforward and versatile approach of polyol synthesis in 1989 to produce colloidal particles of different sizes and shapes composed of metals and alloys. Later, Sun et al. (Sun et al., 2002) and Wiley et al. (Wiley et al., 2005) altered the conventional process in many aspects to create the present approach to polyol synthesis. The polyols, or alcohols with multiple hydroxyl groups like glycerols, ethylene glycols, pentane diols, and so on, reduce the metal salt precursor at higher temperatures, and this is referred to as “polyol synthesis.” The higher boiling points and the temperature-dependent reducing properties of polyols make them highly appropriate for the synthesis of anisotropic nanomaterials. In polyol synthesis, the generally employed stabilizer is polyvinylpyrrolidone (PVP) in order to avoid the colloidal particles from getting agglomerated. Because polyols have a very high dielectric constant and may effectively act as a solvent system for both PVP and metal precursors, it has been proven that this approach works well for synthesizing Ag, Pd, and Pt nanostructures. The existence of heteroatoms like nitrogen and oxygen in the stabilizer can coordinate with the metal atoms formed by polyol reduction, stabilizing the structure formed. Moreover, since PVP interacts with various crystallographic facets of the metallic lattice to varying degrees, this might lead to anisotropic growth of the corresponding nanostructure. Im et al. (Im et al., 2005) prepared monodispersed silver nanocubes by the polyol approach, as shown in Figure 5. A new method to produce silver nanoplates and nanoprisms using a polyol approach with oxyethylated carboxylic acid and glucose has been developed by Titkov et al. (Titkov et al., 2022). Glucose and sodium hydroxide increase the yield of nanoplates and reduce their thickness. The resulting particles were characterized using electron microscopy and XRD. The synthesized nanoparticles have potential applications as metallic fillers in ink and paste formulations for 2D and 3D printing to create functional components and devices.

### 3.5 Photochemical synthesis

**FIGURE 6:**
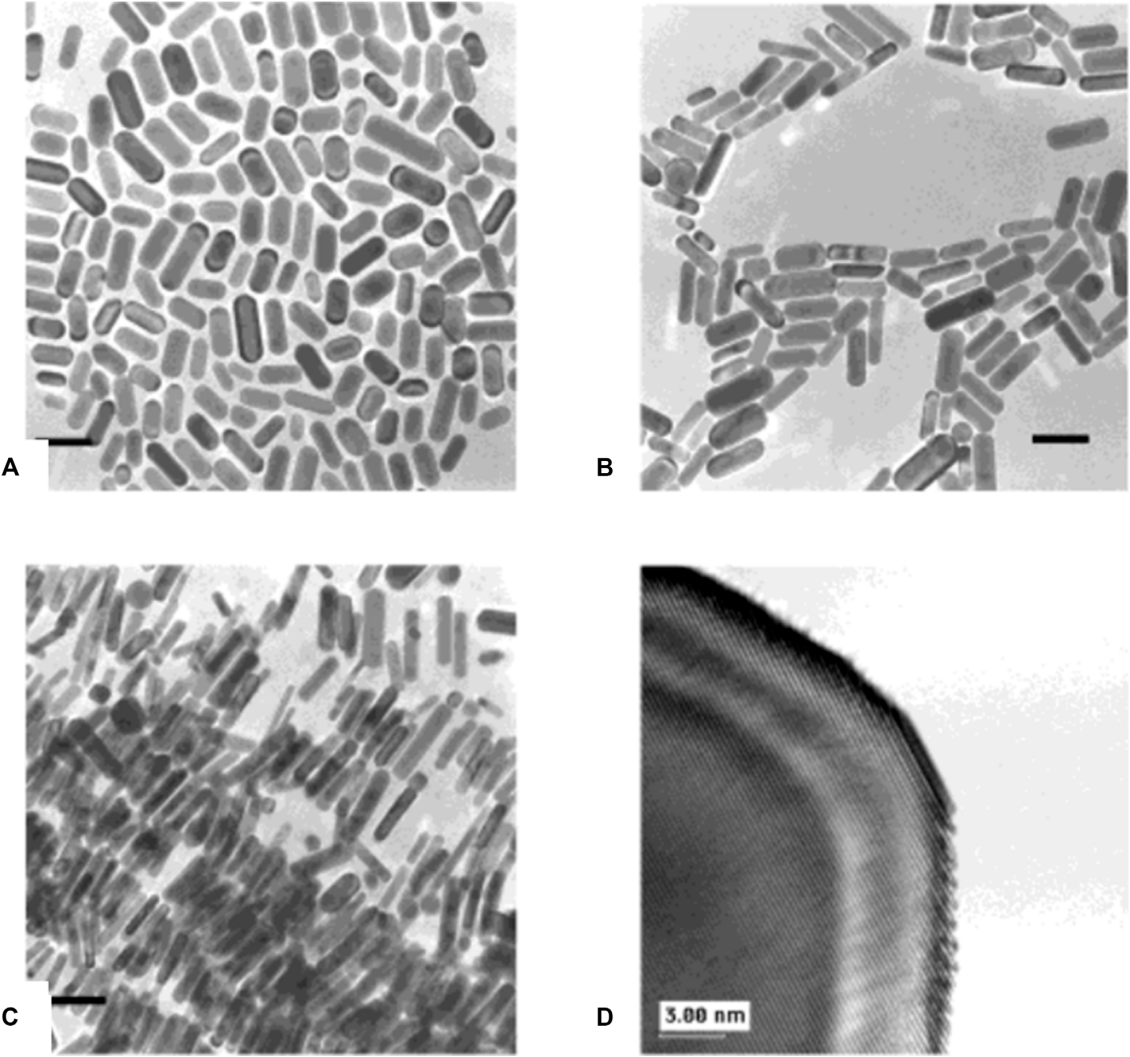
Transmission electron microscopy (TEM) images of gold nanorods prepared with **(A)** 15.8 µL, **(B)** 23.7 µL, **(C)** 31.5 µL of silver nitrate solution. The bar indicates 50 nm. **(D)** High-resolution image of a gold nanorod. [Reprinted with permission from Ref. (Kim et al., 2002), American Chemical Society].

Studies have revealed that radiolytic and photochemical approaches may also be used to reduce metal salt precursors, with the major benefit of not using reducing agents in excess for the reduction process (Esumi et al., 1995; Kundu and Liang, 2008). Here, radiation is absorbed independent of the presence of solute molecules or products that absorb light. Meanwhile, the rate at which reduction reaction takes place is also identified as the amount of reducing equivalents produced by radiation is precisely defined. Also, expensive and complicated equipment is not required in the case of photochemical synthesis. By using UV irradiation, gold colloids with rod-like morphology were synthesized by Esumi et al. (Esumi et al., 1995). Photochemical reduction was applied here for the synthesis of gold nanoparticles from a corresponding gold precursor along with cetyltrimethylammonium chloride micelle, which has a rod-like structure. Since an expansion in the length of NRs simultaneously upsurges the percentage of spherical particles, it appears that this process makes it harder to synthesize homogeneous rods and control the aspect ratio. A photochemical approach proposed by Kim et al. was used to synthesize NRs with homogeneity and a precisely controlled aspect ratio (Figure 6) (Kim et al., 2002). This approach incorporated principles from template-assisted synthesis and is analogous to the electrochemical procedure. Benalcázar et al. looked into a low-cost photochemical reduction method to improve the antibacterial activity of narrow-sized anisotropically flat silver nanoprisms (S-NPs) anchored to graphene oxide (GO) against *Escherichia coli* (Benalcázar et al., 2022). In this study, spherical silver nanoparticles (Ag-NPs) were transformed into S-NPs on GO using a simple, low-cost photochemical reduction process. By adjusting the irradiation wavelength, this photochemical reduction approach allows for precise control of the size and shape of Ag-NPs grown on GO.

### 3.6 Electrochemical synthesis

**FIGURE 7:**
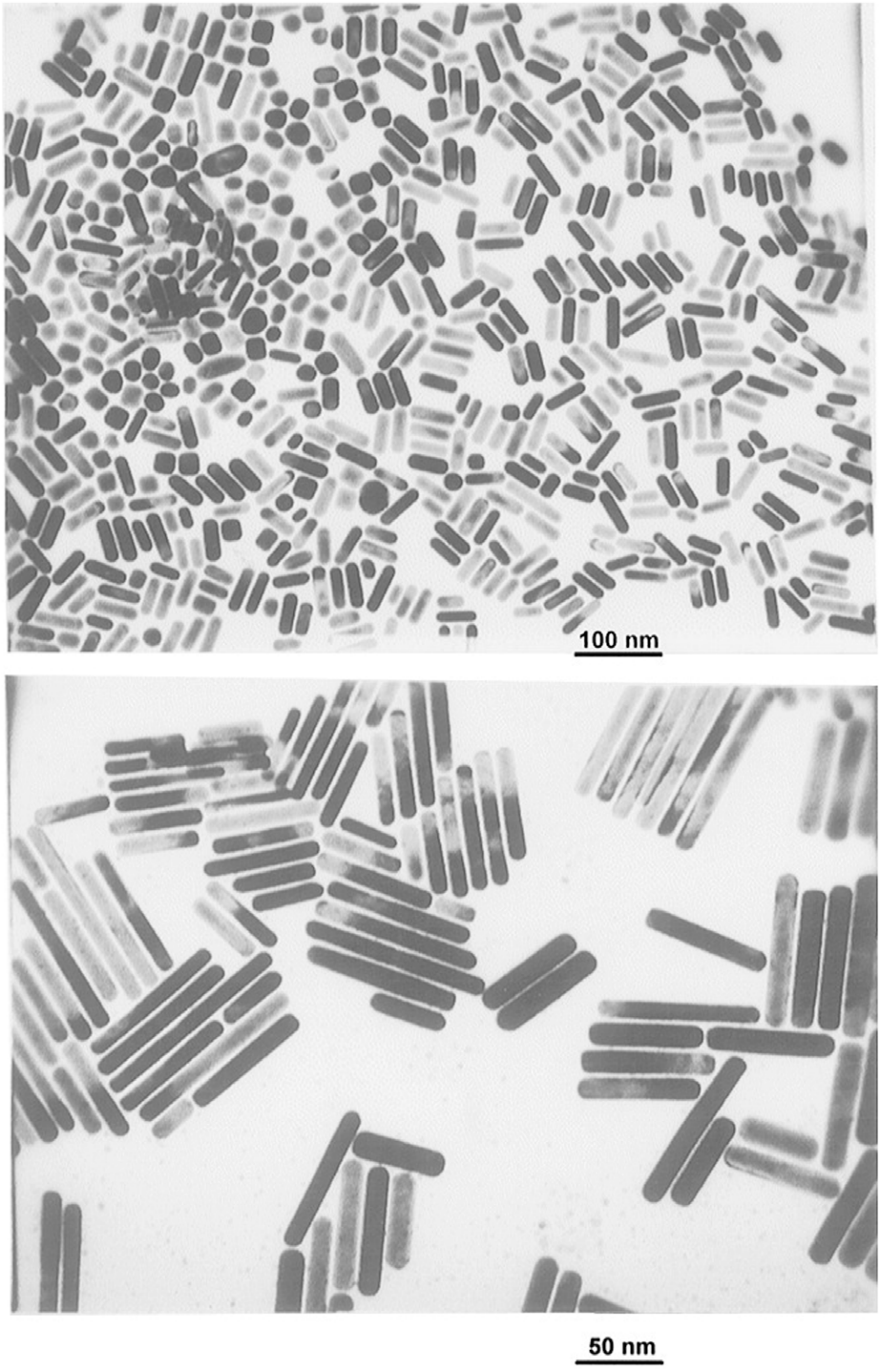
TEM images of Au nanorods with different mean aspect ratios: 2.6 (top) and 7.6 (bottom). [Reprinted with permission from Ref. (Yu et al., 1997), American Chemical Society].

Numerous nanoparticles, particularly those composed of noble metals, have been synthesized using electrochemical techniques (Reetz and Helbig, 1994; Huang et al., 2006). This approach offers several benefits over other methods, including a low operating temperature, good-quality products, the utilization of simple equipment, and being economical. Reetz and Helbig demonstrated in 1994 that by varying the current density, the electrochemical reduction approach may be used to fabricate nanoparticles with high size-selectivity (Reetz and Helbig, 1994). The experimental apparatus includes a two-electrode configuration, with the metal to be converted into a metal colloid that functions as the sacrificial anode. The metal oxidizes at the anode in the whole process. Simultaneously, metal cations move to the cathode along with the reduction that takes place while developing a metal colloid under the influence of a stabilizing agent. Using gold plate as an anode and platinum plate as a cathode, Yu et al. synthesized gold NRs (Figure 7) via an electrochemical approach (Yu et al., 1997).

### 3.7 Hydro/solvothermal synthesis

**FIGURE 8:**
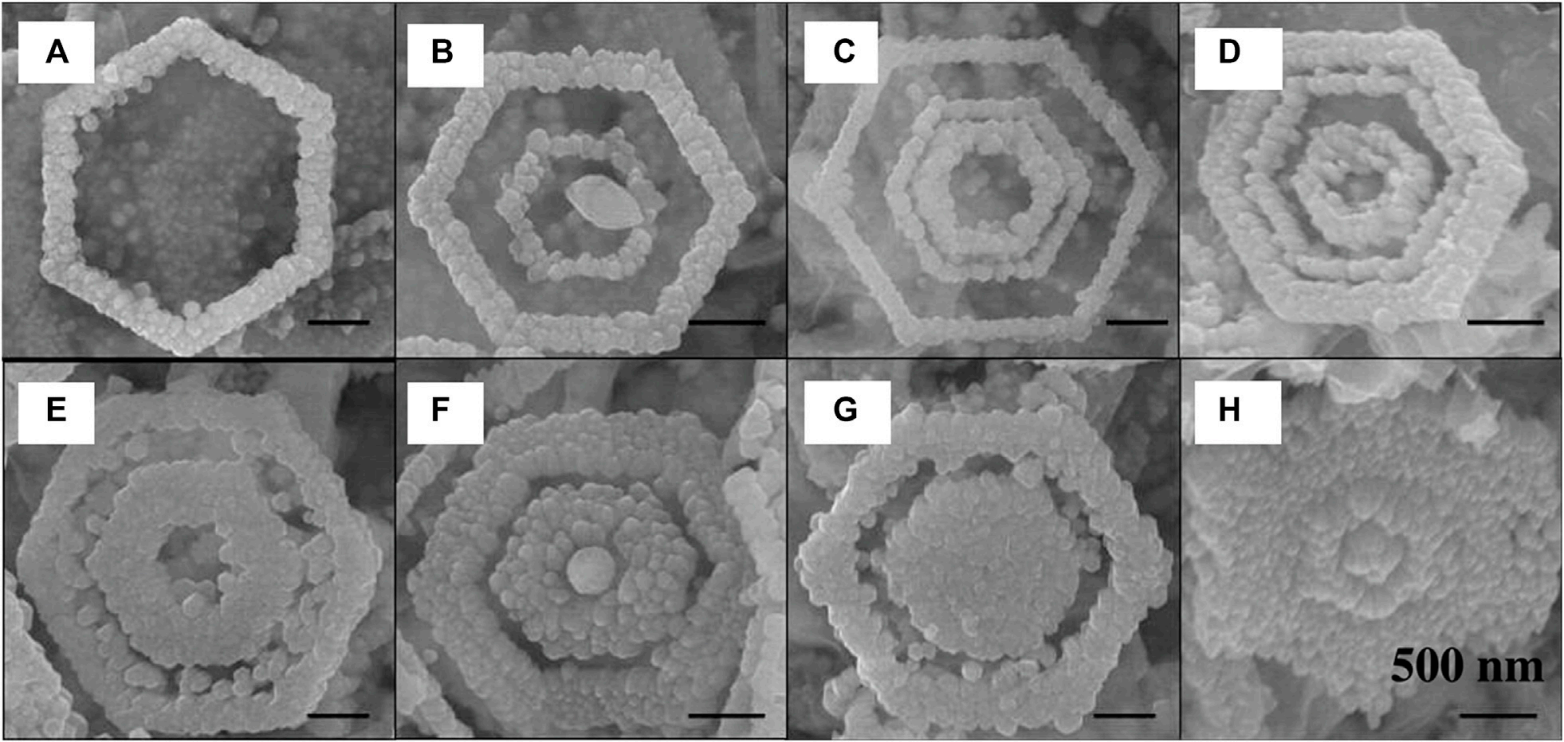
SEM images showing **(A)** single-, **(B)** double-, and **(C)** triple-turn HRLSs, **(D–H)** concentric rings lined up and filled up to different degrees. [Reprinted with permission from Ref. (Li et al., 2008), American Chemical Society].

The hydrothermal or solvothermal synthesis approach uses hot water or a solvent to produce nanoparticles under high pressure in an autoclave (Cansell et al., 1999; Lu et al., 2006). In this method, water functions as a catalyst while also being a rare solid-phase component. As several solvents may be utilized, the synthetic approach is sometimes referred to as solvothermal in line with the overall process concept. Furthermore, additives may be used to alter the original characteristics of pure water. Polar or non-polar solvents may be utilized for the dissolution and recrystallization processes to broaden the scope of this synthesis technique. Hydrothermal approach has been applied for the synthesis of a wide range of materials so far. Hydrothermal synthesis could form multi-layered Co-doped ZnO with a hexagonal ring-like morphology under the influence of an electric field, as demonstrated by Li et al. (Figure 8) (Li et al., 2008). The electric dipole interaction of NRs is critical to the orientation of NRs in this case. This methodology may also be used to synthesize other fascinating structures like nanorings and microloops (Shen and Chen, 2006). Ultrathin 2D titanium dioxide-bronze nanosheets (TiO_2_-BNS) were prepared by following solvothermal synthesis approach using ethylene glycol under green conditions by Rej et al. and were studied for photocatalytic H_2_ evolution during exposure to both UV and solar light irradiation (Rej et al., 2022).

## 4 Anisotropic materials for CO_2_ utilization

The term “CO_2_ utilization” refers to the process of converting carbon dioxide into goods or chemicals that have some economic benefit (Valluri et al., 2022). This process may help alleviate the impacts of having an excessive amount of CO_2_ in the atmosphere. The increased CO_2_ levels in the atmosphere are now generally believed to be directly related to climate shifts. In order to slow down the rate at which carbon emissions are rising, it is crucial to switch to renewable energy sources that are not carbon-based. But looking at the current technological scenario, it is impossible to make such a switch over swiftly, and we may have to depend on fossil fuels for our energy requirements for quite some time. The chemical processes involving CO_2_ are unfavorable thermodynamically due to the fact that carbon at its maximum oxidation state is highly inactive (Schneider et al., 2012). Because of this, employing CO_2_ as a feedstock is complicated, and hence the process for CO_2_ utilization should be developed in such a way that all these hurdles are looked into. Recent studies involving anisotropic materials for effective CO_2_ utilization, are promising wherein these anisotropic materials are effectively utilized in photocatalytic, thermocatalytic, and electrocatalytic pathways which are tabulated in Table 1. Table 2 depicts different CO_2_ reduction products and their associated thermodynamic potentials at pH 7 in an aqueous solution against a conventional hydrogen electrode (NHE) at 25°C and 1 atm pressure (Nguyen et al., 2020; Jin et al., 2021; Rej et al., 2021).

**TABLE 1:** Table showing anisotropic nanomaterials with different morphologies and their corresponding activity in CO_2_ utilization.

Sl. No.	Catalyst	Morphology	Catalytic activity	Reference
1	WO_3_	Quasi-cubic-like	Oxygen evolution reaction and CH_4_ generation	Xie et al. (2012)
2	MnO_x_ and Pt deposited single-domain ferroelectric PbTiO_3_ nanoplates	Nanoplate	Solar energy conversion	Zhen et al. (2014)
3	Cu_2_O crystals	Edge-truncated cube	Methanol production by CO_2_ photoreduction	Kumar Sahu et al. (2022)
4	Co-coordinated g-C_3_N_4_ layer and Au NRs nanocomposite	Nanorod	CO generation by CO_2_ photoreduction	Yoshii et al. (2021)
5	ZnSe/CdS dot-on-rod (DOR) nano-heterostructure	Rod	Artificial photosynthesis	Xin et al. (2022)
6	AgX:Ag (X = Cl, Br) nanoparticles/nanoplates	Cube-tetrapod-like	Methanol production by CO_2_ photoreduction	An et al. (2012)
7	Ag^0^ particles loaded H_2_SrTa_2_O_7_	Cubic (Ag^0^ particles)	CO generation by CO_2_ photoreduction	Wang et al. (2019)
8	Cu_2_O/rGO	Rhombic dodecahedra	Methanol production by CO_2_ photoreduction	Liu et al. (2019)
9	xCe/Ni@SiO_2_	Nanotube	Dry reforming of methane	Zhou et al. (2022)
10	Ni/CeZrO_2_	NA	Dry reforming of methane	Dai et al. (2021)
11	Pd/ZnO catalyst	Hexagonal plate-like	Methanol production by CO_2_ reduction	Zhang et al. (2021)
12	Au nanosheets	Nanosheet	Electrochemical reduction of N_2_ and CO_2_ into urea	Bharath et al. (2022)
13	Cu and Fe-based electrocatalysts	Dendritic-type nano-morphology	Formic acid synthesis by electroreduction of CO_2_	Marepally et al. (2020)
14	Tri-Ag-NPs	Ag nanoplates with triangular shape	CO generation by CO_2_ electroreduction	Liu et al. (2017)

**TABLE 2:** Products with corresponding thermodynamic potentials derived from CO_2_ reduction.

Reaction	Product	E^o^ (V vs. NHE)
CO_2_ + e^−^ CO_2_^.-^	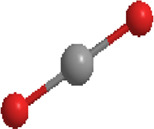 Carbon dioxide radical anion	−1.90 V
CO_2_ + 2H^+^ + 2e^−^ HCOOH	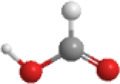 Formic acid	−0.61 V
CO_2_ + 2H^+^ + 2e^−^ CO + H_2_O	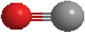 Carbon monoxide	−0.53 V
CO_2_ + 4H^+^ + 4e^−^ HCHO + H_2_O	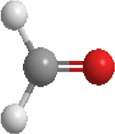 Formaldehyde	−0.48 V
CO_2_ + 6H^+^ + 6e^−^ CH_3_OH + H_2_O	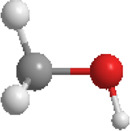 Methanol	−0.38 V
CO_2_ + 8H^+^ + 8e^−^ CH_4_ + 2H_2_O	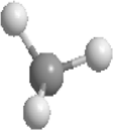 Methane	−0.24 V

### 4.1 Photochemical CO_2_ utilization

In recent years, CO_2_ reduction by photocatalysis for the production of fuels and other value-added chemicals has gained a great deal of interest due to the fact that it presents a green approach for the effective utilization of CO_2_ and that it addresses carbon reduction. The formation of electron–hole pairs by the photocatalyst by absorbing light is the first step in the photocatalytic CO reduction, which is followed by charge migration, the oxidation of water, and a reduction reaction of CO_2_ on the surface of the photocatalyst (Liu et al., 2011; Low et al., 2017; Patil et al., 2019). Improving the photocatalytic activity requires first figuring out how to effectively separate and move the photogenerated charge carriers. It is necessary for electron-hole pairs to move to the active sites, before they can be recombined in order to obtain a higher conversion efficiency, on the surface of the catalyst. However, owing to the robust Coulomb’s electrostatic pull, there is the possibility of recombining of electron-hole pairs as they migrate to surface of the catalyst. Recombination is caused, in part, by the closeness between locations on the surface that accumulate electrons and sites that accumulate holes, as well as by the absence of defined pathways for the electrons and holes to transfer (Xu and Carter, 2018). For this reason, a comprehensive knowledge of the dynamics of photogenerated charge carriers is vital for the logical design of a photocatalyst that is active and efficient. Recent research has shown that a spatial charge separation can be achieved amongst the different anisotropic facets, and it is believed that this charge separation between anisotropic facets of the crystals is responsible for the improved photocatalytic performance, which is achieved by allowing photogenerated charge carriers to participate in redox reactions more effectively. The preferential arrangement of active reduction and oxidation sites on anisotropic facets is also due to the spatial separation of charge carriers (Chen et al., 2018). This efficiently reduces charge carrier recombination, resulting in an increase in CO_2_ conversion efficiency. As a result, designing the facets of the crystal is a crucial component in order to ensure effective charge separation.

**FIGURE 9:**
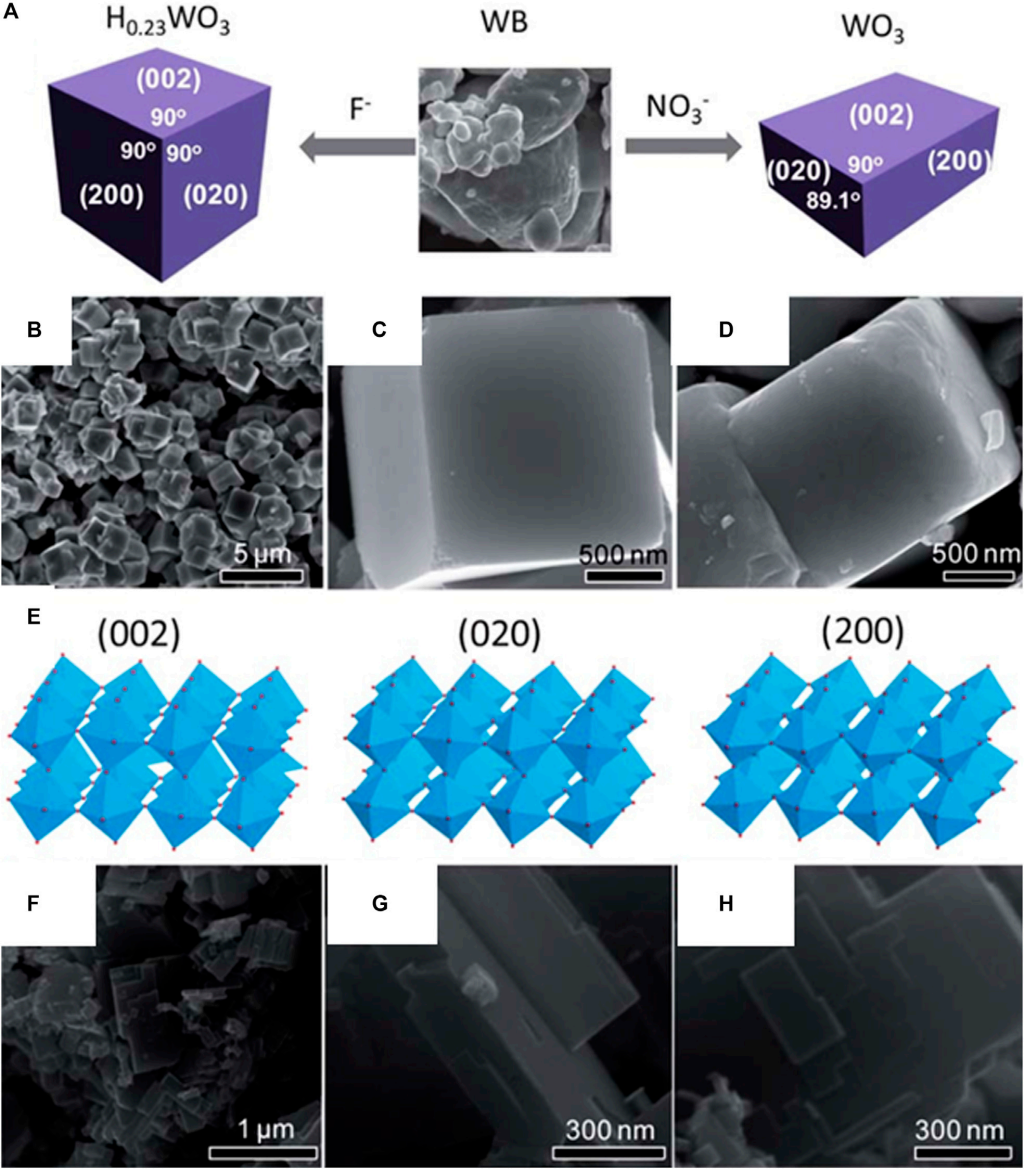
**(A)** Schematic of the growth of a cubic H_0.23_WO_3_ crystal and rectangular sheet-like monoclinic WO_3_ crystal from WB precursor in the presence of HF and HNO_3_ aqueous solutions; **(B)** and **(C)**, SEM images of cubic H_0.23_WO_3_ crystals; **(D)**, SEM image of quasi-cubic-like WO_3_ crystals by calcining the cubic H_0.23_WO_3_ crystals; **(E)**, atomic structure models of (002), (020) and (200) facets in a monoclinic WO_3_; **(F–H)**, SEM images of rectangular sheet-like WO_3_ crystals. [Reprinted with permission from Ref. (Xie et al., 2012), Royal Society of Chemistry].

By controlling the acidic hydrolysis of crystalline tungsten boride (WB), Xie et al. were able to report a simple and novel method for producing a quasicubic-like WO_3_ crystal with an almost similar percentage of {002}, {200}, and {020} facets, as well as a rectangular sheet-like WO_3_ crystal with a predominant {002} facet (Figure 9) (Xie et al., 2012). The photocatalytic activity of the catalyst depends on a specific surface area and may be optimized for specific reactions owing to the difference in electronic band edges between the two kinds of WO_3_ crystals, which is induced by the crystal facet electronic structure effects. The quasi-cubic-like WO_3_ crystal with a larger valence band maximum exhibits significantly greater O_2_ evolution rates in photocatalytic water oxidation than the rectangular sheet-like WO_3_ crystal because of electronic structural features brought on by crystal facets. In the presence of water vapor, which has a higher conduction band minimum of 0.3 eV, CO_2_ can be photoreduced to produce CH_4_. The quasi-cubic WO_3_ crystals have an oxygen evolution rate that is 8.4 times greater than the rectangular sheet-like crystals when normalized by specific surface area. The increased oxidative power of photoexcited holes produced from the lower valence band (VB) maximum is responsible for the considerably higher oxygen evolution rate of the quasi-cubic-like crystals, the reason being the larger specific surface area of the quasi-cubic-like crystals of WO_3_ when compared to that of the rectangular sheet-like crystals.

According to research carried out by Zhen et al., using a ferroelectric field, it is possible to deposit the noble metals and their metal oxides on the positively and negatively charged {001} facets of PbTiO_3_ nanoplates (Zhen et al., 2014). The H_2_ generation with Pt particles on PbTiO_3_ that were preferentially placed on the positively charged {001} facet was enormously more efficient compared to the photocatalytic H_2_ evolution on PbTiO_3_ with Pt particles that were indiscriminately deposited. The photocatalytic activity of the products obtained in this way is much higher than that of the products obtained by randomized deposition. This proposed method has the potential to be beneficial in the advancement of photocatalysts for solar energy conversion that are based on ferroelectric materials and have high efficiency.

**FIGURE 10:**
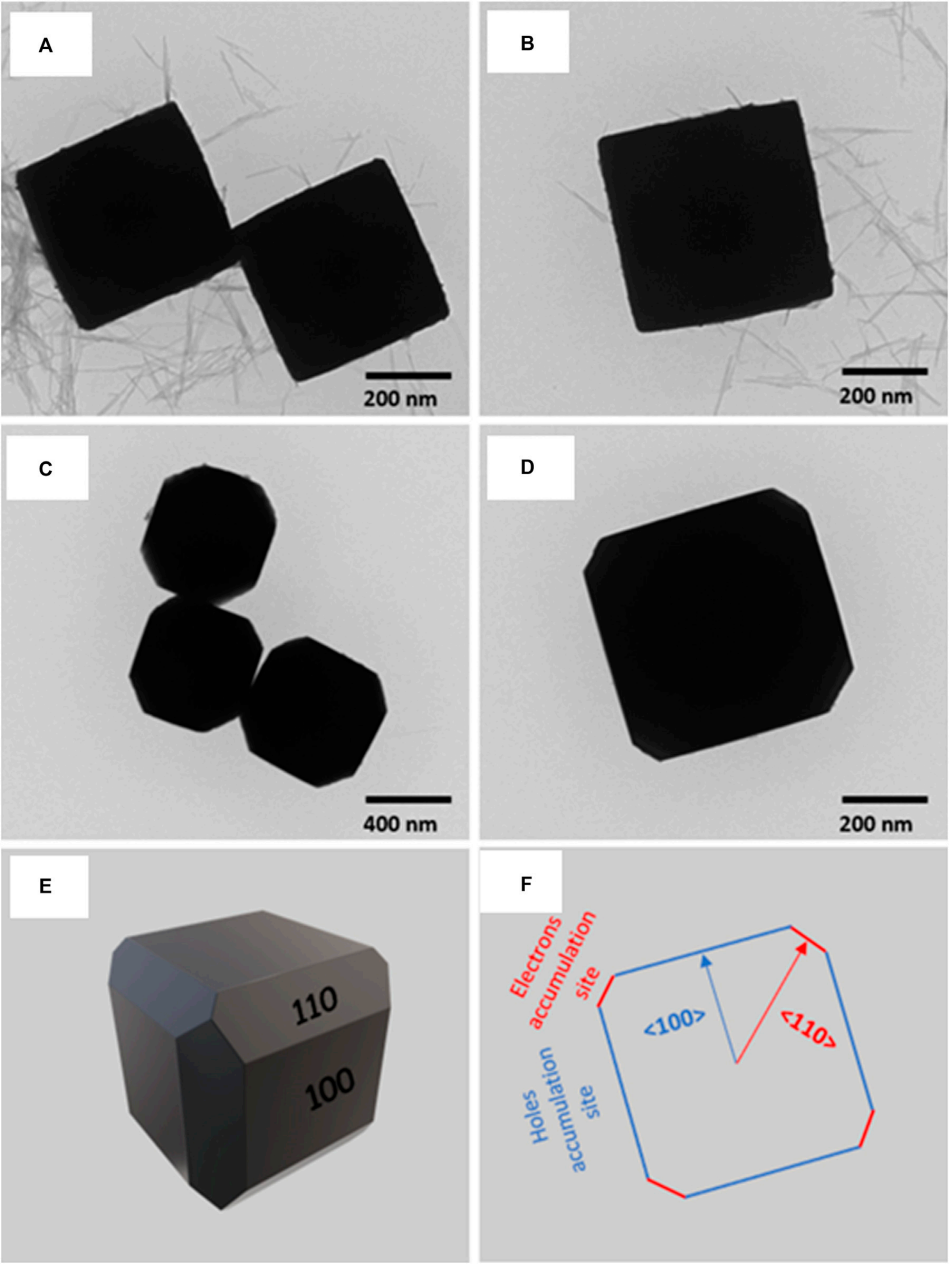
TEM images of Cu_2_O microcrystals of different morphologies: **(A, B)** cubes and **(C, D)** edge-truncated cubes. **(E)** Simulated 3D structure of the edge-truncated cubic Cu_2_O microcrystal and **(F)** 2D crystal orientation. [Reprinted with permission from Ref. (Kumar Sahu et al., 2022), American Chemical Society].

According to the findings of computational simulations that Kumar Sahu et al. conducted on Cu_2_O crystals with various facets, edge-truncated cubic Cu_2_O showed to permit effective charge separation (Kumar Sahu et al., 2022). Cu_2_O photocatalysts having two separate morphologies and facet alignments, namely, cubic and edge-truncated cubic structures, were prepared and studied in order to validate the computational predictions (Figure 10). It was found that the photocatalytic activity toward the preferential CO_2_ reduction to form methanol on the edge-truncated cubic Cu_2_O with anisotropic {100} and {110} facets was found to be almost 5.5 times greater compared to the cubic Cu_2_O with just {100} facets. This difference in behavior can be explained by the efficient separation and migration of photogenerated charge carriers, in addition to the selective aggregation of electrons and holes on various facets of edge-truncated cubic Cu_2_O crystals. In addition to this, the impacts of the changes in work function between the {110} and {100} facets on the electronic band structure and the anisotropic charge separation were revealed. These results give crucial guidance for the development and fabrication of highly effective and precise photocatalysts for the transformation of CO_2_ into fuel.

Yoshii et al. demonstrated a novel kind of nanocomposite catalyst having a core-shell structure wherein catalytically active Co single-site species are anchored on graphitic carbon nitride (g-C_3_N_4_) coated on gold NRs for SPR-intensified photocatalytic conversion of CO_2_ (Yoshii et al., 2021). A simple pyrolysis technique was used to integrate single-site Co species into g-C_3_N_4_, and nanocomposites with core-shells subsequently self-structured through electrostatic interactions amongst the surfaces of the Au NRs, which are positively charged, and the single-site Co-coordinated g-C_3_N_4_ layer, which is negatively charged. Analyses were used to thoroughly study the structure, which revealed that the g-C_3_N_4_ layer did not inhibit the Au NRs from absorption of visible light. With the help of the Au NRs’ SPR, the nanocomposite effectively accelerated the CO_2_ reduction to form CO with visible light irradiation. The core-shell-structured catalyst outperformed the similar aggregated catalyst in terms of catalytic activity, suggesting that the development of a core-shell structure allows for effective electron transportation from the Au NRs to the active Co species.

Xin et al. have reported the successful usage of a well-constructed ZnSe/CdS dot-on-rod (DOR) nano-heterostructure using H_2_O as an electron donor for the CO_2_ reduction photocatalytically (Xin et al., 2022). The DOR nano-heterostructure is formed in a manner that is distinct from the more conventional core-shell or dot-in-rod (DIR) nano-heterostructures. This is accomplished by anchoring numerous ZnSe QDs to the surface of a single CdS nanorod. Studies using steady-state and time-resolved spectroscopy provide insight on the dynamics of charge carriers, in particular the transfer of holes across interfaces. Intuitively, surface photovoltage microscopy verifies that holes congregate on ZnSe while electrons disperse across the CdS domain. ZnSe/CdS DORs display far greater activity (11.3 μmol g^-1^h^-1^) and selectivity (85%) to CO_2_ to CO photoreduction than that of pristine CdS nanorods or ZnSe/CdS DIRs under similar circumstances. This is because of the ultrafast charge separation of electrons and holes, as well as the good exposure of holes on the surface. It was found that the ZnSe/CdS DOR nano-heterostructure is extremely stable, since the photocatalytic activity did not alter after 80 h of experimentation.

**FIGURE 11:**
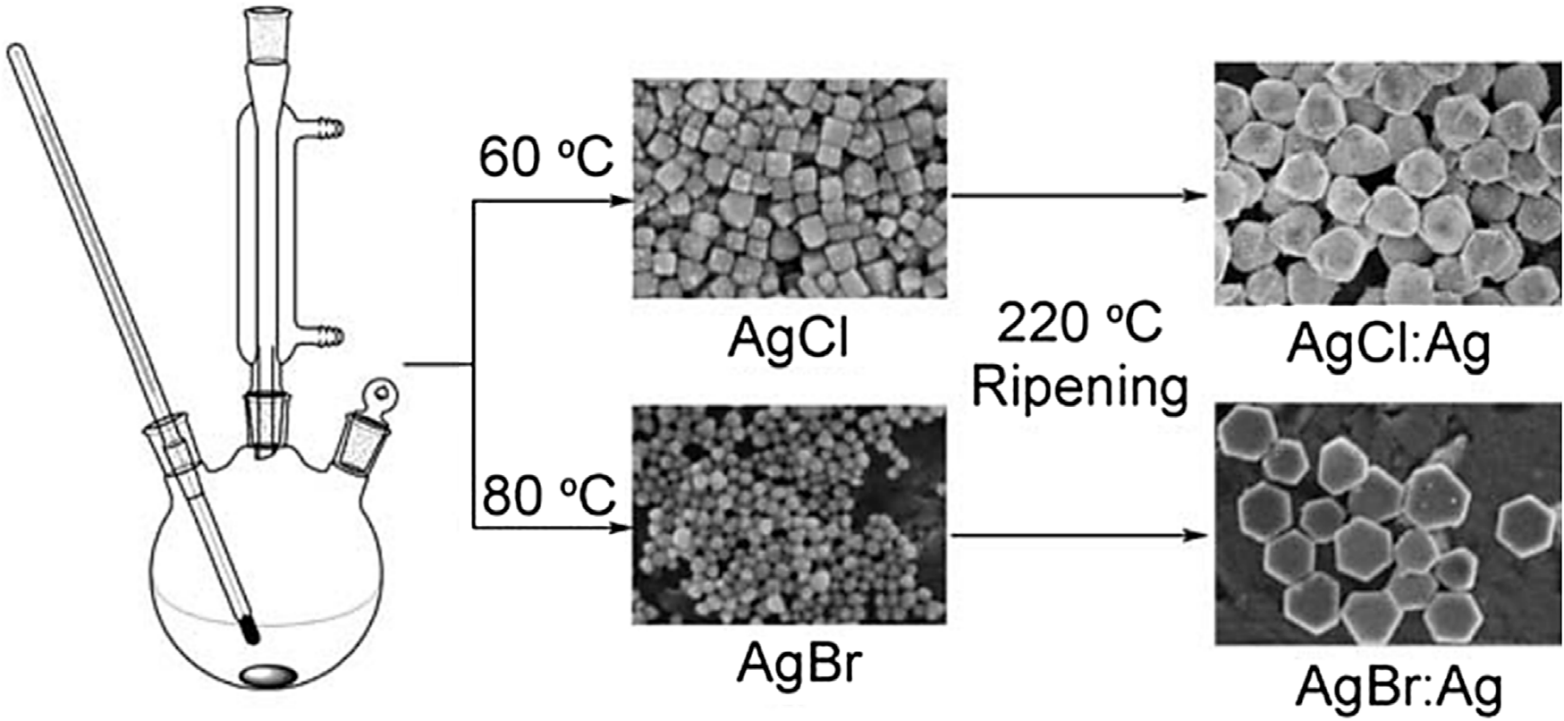
Schematic illustration of the synthesis of AgCl:Ag and AgBr:Ag nanoparticles. [Reprinted with permission from Ref. (An et al., 2012), Royal Society of Chemistry].

An et al. prepared plasmonic-shaped AgX:Ag (X = Cl, Br) nanoparticles by a simple and adaptable glycerol-mediated solution process (as shown in Figure 11), and the prepared AgX:Ag nanoparticles have regular shapes, like cube-tetrapod-like AgCl:Ag nanoparticles and AgBr:Ag nanoplates (An et al., 2012). The as-prepared AgX:Ag nanoparticles exhibit regular shapes, i.e., cube-tetrapod-like AgCl:Ag nanoparticles and AgBr:Ag nanoplates. Because of the SPR of silver nanoparticles, AgX:Ag nanocomposites absorb more light in the visible area than pure AgX. From the bandgap calculations and band sites, it is revealed that the AgX:Ag nanoparticles may be employed as a potential photocatalyst for CO_2_ reduction. The precipitation reaction between AgNO_3_ and NaX in glycerol with the help of PVP produced uniformly sized AgX nanoparticles. At lower temperatures, the viscosity of glycerol is high, and at the same time, at higher temperatures, glycerol has strong reducibility, which makes glycerol an ideal reaction medium for the synthesis of evenly shaped AgX:Ag. Because of the SPR of silver nanoparticles, the as-prepared AgX:Ag nanophotocatalysts displayed substantial absorption in the visible range and a strong capacity to convert CO_2_ to methanol. Also, there was no discernible reduction in photocatalyst activity during recycling processes.

For the objective of photocatalytic CO_2_ reduction, layered perovskite material H_2_SrTa_2_O_7_ (HST) with excellent charge separation was synthesized by Wang et al. using polymerizable complex (PC) and ion-exchange techniques (Wang et al., 2019). Ag particles were placed on HST as a co-catalyst in order to create the active sites for CO_2_ reduction. This was done as a means of overcoming the problem of poor selectivity. Due to the structural anisotropy of the layered perovskite material, the Ag metal (Ag0) particles were placed on the edge of HST in a selective manner, which helped to maintain a clean separation of reduction and oxidation sites in space. Ag particles have the ability to lower the overpotential of CO evolution and encourage more electrons to be emitted from HST for the purpose of reducing CO_2_ to CO rather than H^+^ to H_2_, which in turn helps to promote selectivity toward CO evolution. The photocatalyst with 0.5 weight percent Ag displayed the highest selectivity for carbon monoxide (60.9%), which is 2.1 times higher than that of pure HST (28.5%). This study offers some pointers on how to prepare photocatalysts that have a high selectivity for carbon compounds resulting from the reduction of CO_2_.

The fabrication of cuprous oxide (Cu_2_O) having various shapes and oxidation states, decorated by reduced graphene oxide (rGO), and the assessments of their photocatalytic CO_2_ reduction performance were studied by Liu et al. (Liu et al., 2019). After 20 h of exposure to visible light, the rhombic dodecahedra Cu_2_O/rGO yielded a methanol output of 355.3 μmol g^-1^cat, which is the highest when compared to the cubic and octahedral Cu_2_O/rGO and CuO/rGO structures. The improved performance may be attributable to the one-of-a-kind structure of rhombic dodecahedra, which has reduced band bending of the conduction and valence bands. This reduces the energy barrier for the photogenerated electrons to transit to the surface, which contributes to the improved performance. It is anticipated that the presence of rGO would facilitate the transport of photogenerated electrons from the conduction band of Cu_2_O. It is possible that the positively charged dodecahedra Cu_2_O/rGO will boost the adsorption of carbonate anions that result from the dissociation of CO_2_ gas. In order to fabricate crystals of Cu_2_O with rGO integrated that are visible-light active photocatalysts for the use of CO_2_ utilizing solar energy, this study offers a simple solution-chemistry approach.

#### 4.2 Thermochemical CO_2_ utilization

In the absence of a realistic and financially viable solution to the CO_2_ catastrophe, the consumption of fossil fuels will be curtailed by the increase in CO_2_ levels and the environmental concerns that accompany it. Therefore, the optimal solution can be as simple as capturing the CO_2_ from any source, including the atmosphere, and recycling it into fuels and value-added chemicals. A significant transition of the global energy base away from fossil fuels and toward renewable energy and cleaner fuels like hydrogen, the development of practical and viable CO_2_ capture and storage technologies, and the effective utilization of the CO_2_ are some of the strategies that can be practiced for the decarbonization process. Despite its importance, shifting the energy base away from fossil fuels has significant drawbacks, including massive changes in energy systems in the logistics sector as well as political challenges in making these changes in regions with abundant fossil fuel reserves (Höök and Tang, 2013). At the same time, CO_2_ storage has drawbacks such as a high cost and large energy requirements for the process, and the permanent storage of CO_2_ at sites will be difficult (Al-Mamoori et al., 2017). Hence, the method of carbon utilization via CO_2_ conversion is highly efficient when compared to sequestration and is the most realistic and sustainable approach to slowing down climate change while reducing anthropogenic emissions. Various studies have been undertaken in order to determine the quantity of CO_2_ that must be captured and transformed in order to have a significant influence on climate change (Wennersten et al., 2015). These studies were primarily focused on methods to decrease CO_2_ emissions from the non-renewable energy system quantitatively. But when the CO_2_-converted fuels are subjected to combustion, then it creates an equivalent quantity of CO_2_, resulting in no direct net CO_2_ consumption. Being a thermodynamically stable, highly oxidized molecule with two linear double bonds and very low reactivity, the activation of CO_2_ requires breaking through a thermodynamic barrier, which makes the chemical conversion and economical use of CO_2_ a difficult scientific and technological challenge (De et al., 2020).

There are currently just a handful of industrial processes that make use of CO_2_, such as the production of polycarbonates, urea, and salicylic acid (MacDowell et al., 2010; Porosoff et al., 2016; Alper and Orhan, 2017). The hydrogenation reaction is one kind of reaction that has been recognized as the most significant of the many CO_2_ chemical transformations because it provides a great opportunity for sustainable growth, especially in the fields of energy and environment. Due to its high thermodynamic stability, the reduction of CO_2_ is an energy-intensive process requiring suitable reductants and effective catalysts. In comparison to other methods, thermocatalytic hydrogenation stands out due to its scalability, adaptability to the existing industrial setup, and broad product range. To address the critical challenges involved in the thermocatalytic reduction of CO_2_, a comprehensive understanding of each step involved is required, along with a rational catalyst design and energy optimization brought about by the innovative reactor design. In addition, the products obtained from the conversion of CO_2_ can be considered value-added since they may be used as fuels or as precursors in the production of other complex chemicals and fuels. The Sabatier reaction was the first reaction in which methane was prepared industrially by the hydrogenation of CO_2_ (Vogt et al., 2019; Ooka et al., 2021). This discovery has been a crucial step in the process of comprehending the fundamental principles that underlie contemporary catalysis. However, once the Fischer–Tropsch (FT) method was developed for the synthesis of hydrocarbons from syngas, the Sabatier reaction lost its relevance in the industrial sector (Gholami et al., 2021). In terms of stability and reactivity, CH_4_ is much more stable and less reactive when compared to methanol when it comes to the formation of subsequent derivatives and chemicals. This makes CH_4_ the simplest C1 hydrocarbon of CO_2_ conversion.

In spite of all of these efforts, it is quite obvious that the technology for CO_2_ hydrogenation is still far from widespread commercialization, which is vital in order to produce the anticipated change in the environment. This is largely the result of poor conversion and output selectivity, both of which are brought about by variables that are both competitive and unfavorable from a kinetic and thermodynamic perspective. The way forward to solve these problems is to design and develop catalysts and integrated reactor systems that are more effective and selective and that are capable of producing the required products with high conversion rates while incurring the lowest possible energy costs. To be able to design better catalysts, one must have an in-depth grasp of the reaction processes that are the gold standard. However, defining reaction processes in the case of thermochemical hydrogenation of CO_2_ is tough and problematic since there is a lack of strong *in situ* probing tools, the complexity of extreme reaction conditions, and reactor designs that are spectroscopically opaque. Therefore, currently efforts are being put into research and development of CO_2_ hydrogenation, and there has been progress made in synthesizing highly active and efficient catalysts with enhanced selectivity and stability for thermochemical CO_2_ reduction based on the mechanistic knowledge involved in the catalytic processes. As a result, anisotropic materials have the potential to form an interesting class of materials in this respect; nevertheless, a significant amount of study has to be conducted specifically in this area. In spite of the fact that research is being conducted in this area, the thermocatalytic study of anisotropic nanomaterials in CO_2_ utilization is not well explored.

Zhou et al. devised a coating technique that 100% confines Ni nanoparticles inside SiO_2_ nanotubes (NTs), demonstrating superior Ni sintering resistance for dry reforming of methane (DRM) (Zhou et al., 2022). In order to increase catalytic stability and elucidate the reaction mechanism, xCe/Ni@SiO_2_ catalysts with varying Ce loadings were prepared by first loading CeO_2_ onto the surface of Ni phyllosilicate NTs (Niphy NTs), followed by a coating with a layer of SiO_2_. Because of its high oxygen vacancies (Vo) and turnover frequency, the catalyst with a 20% Ce loading demonstrated the highest catalytic performance for DRM. Furthermore, in the 80 h stability study, it demonstrated steady conversions of CH_4_ and CO_2_ (76.3% and 80.1%, respectively), with a weight loss of carbon of 17.9%. The reaction mechanism of CH_4_ and CO_2_ is demonstrated using *in-situ* diffuse reflectance infrared Fourier transform spectroscopy (DRIFTS). On the active site, CH_4_ is first cleaved into CHx* and H*, and then H* quickly activates CO_2_ to form the intermediate carbonate species that is subsequently degraded on the surface of CeO_2_ to CO and H_2_O. During the same time, carbon residues generated on Ni during the cracking of CH_4_ may directly react with mobile oxygen to produce an additional CO molecule. Also, CO_2_ may be readily activated on CeO_2_ to produce CO* and O*. This research emphasizes the development of Ni-based bimetallic catalysts contained inside SiO_2_ NTs with excellent sintering resilience as well as other characteristics such as high Vo concentration for use in other reactions at higher temperatures.

In the synthesis of methane by CO_2_ reforming, ceria-zirconia can be a good support for nickel catalysts. A recent study by Dai et al. demonstrated a way to significantly enhance the efficiency of the Ni catalyst supported on the solid solution of ceria-zirconia by decomposing the nickel salt precursor in a dielectric barrier discharge (DBD) plasma at around 150°C, after which a reduction reaction employing hydrogen at 500°C was carried out without DBD plasma (Dai et al., 2021). Plasma decomposition, as a quick operation at low temperature, not only maintains a greater specific surface area of Ni/CeZrO_2_, but also favorably regulates the size of the Ni NPs and the crystallographic planes of the catalyst. The Ni nanoparticles synthesized via plasma decomposition possessed smaller particles with mostly accessible Ni(111) lattice fringes than the catalyst produced by traditional thermal decomposition. Enhanced reducibility in the plasma-decomposed catalyst contributes to the formation of more efficient Ni(0) particles subsequent to reduction by hydrogen. Moreover, it shows more oxygen vacancies and basic sites, which encourage CO_2_ activation. Additionally, this catalyst has a tendency to produce extremely reactive carbon during the breakdown of methane, the primary reaction of CO_2_ reforming, and the subsequent reaction with carbon dioxide. This also increases the resistance to coke formation during CO_2_ reforming.

**FIGURE 12:**
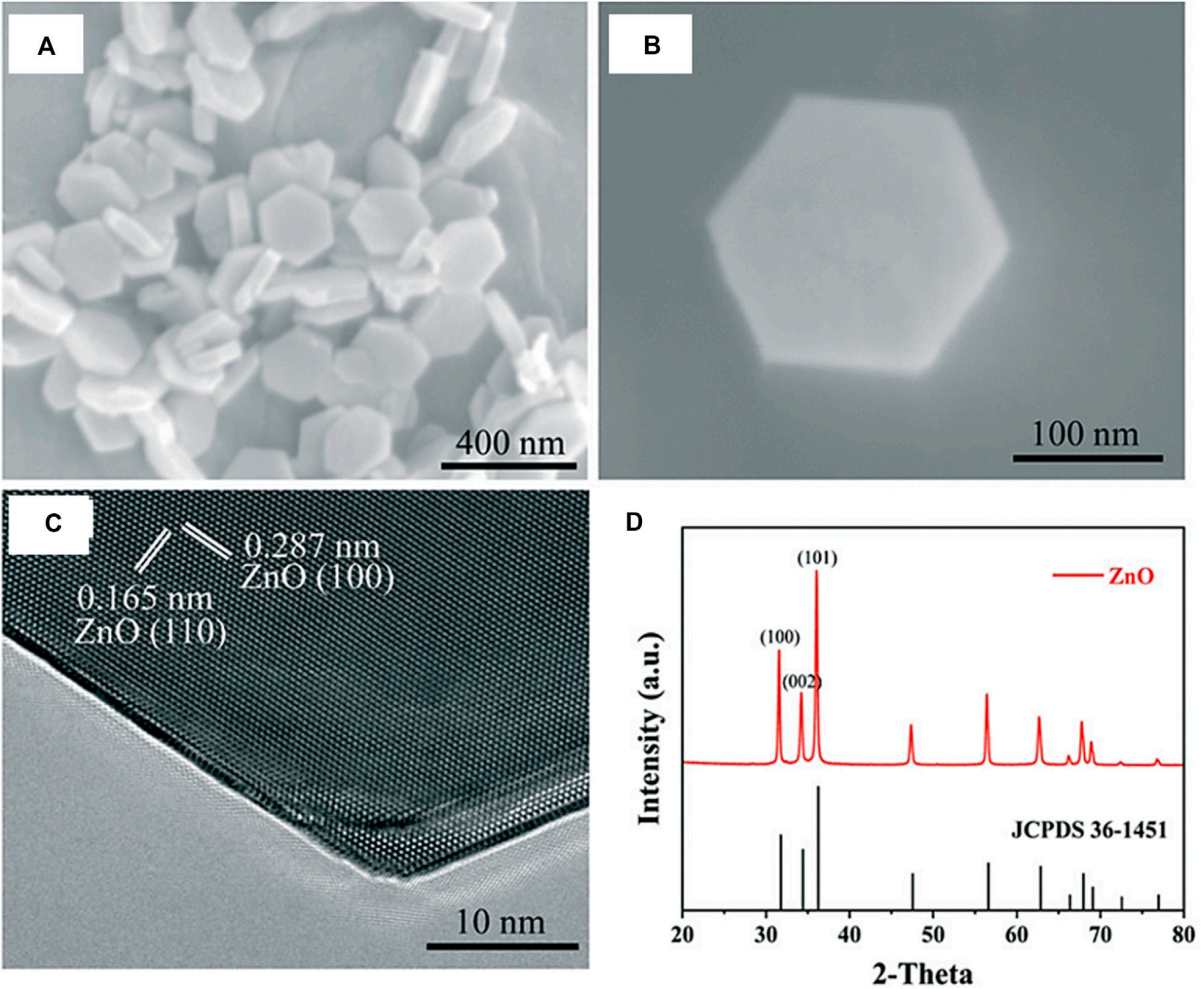
**(A, B)** SEM images of the ZnO nanoplates. **(C)** HRTEM image of ZnO in the [002] view. Two sets of lattice fringes of 0.165 nm and 0.287 nm are identified corresponding to the (110) and (100) planes of wurtzite ZnO, respectively. **(D)** XRD pattern (red) of the ZnO nanoplates. All the diffraction peaks with 2θ of above 30° can be perfectly indexed to the hexagonal phase of wurtzite ZnO (JCPDS card No. 36-1451). [Reprinted with permission from Ref. (Zhang et al., 2021), Royal Society of Chemistry].

In catalytic processes, metal-support interactions have been researched for years. The stability, activity, and selectivity of the catalysts may all be enhanced by properly tuning the metal-support interactions. In a study carried out by Zhang et al., a variety of Pd/ZnO catalysts with different Pd particle sizes were synthesized by the atomic layer deposition (ALD) method (Figure 12) (Zhang et al., 2021). These Pd/ZnO catalysts exhibited metal-support interactions effect depending on the size of Pd particles where during H_2_ reduction at high temperatures, with the bigger Pd particles being more likely to be enveloped by ZnO than the smaller sized ones. Here, it has been demonstrated that during the course of CO_2_ hydrogenation, the Pd particles with sizes bigger than 2.5 nm displayed a greater efficiency and selectivity towards methanol production due to the size-dependent metal-support interactions.

#### 4.3 Electrochemical CO_2_ utilization

For decreasing atmospheric concentrations of CO_2_ and deriving value-added chemicals and fuels via the use of renewable energy sources, electrochemical reduction is an effective approach (Rasul et al., 2015; Sarfraz et al., 2016; Kim et al., 2017; Cao et al., 2018; Zhang et al., 2019; Zhang et al., 2020). Large-scale applications are hampered by significant challenges connected with the efficiency and selectivity of electrocatalysts during the synthesis of multi-carbon products, notwithstanding the significant successes that have been gained so far (Elouarzaki et al., 2019; Sánchez et al., 2019). In order to attain progress in the design and development of efficient and active electrocatalysts, the catalytic mechanisms should be thoroughly understood. This part of the review highlights the most recent developments in the design and development of anisotropic nanomaterials that are efficient in electrocatalytic processes involving CO_2_ utilization. There are a number of benefits that the electrochemical reduction of CO_2_ can offer, which include reaction steps that are controllable, comparatively moderate reaction conditions, and better conversion. Additionally, renewable energy sources such as wind, solar, and hydroelectric energy can be used as a driving power for the reduction reaction effectively and sustainably (Faggion Jr et al., 2019). Despite the fact that the faradaic efficiency may surpass 90%, this technique is hampered by its meager stability in the long term, which can be improved by synthesizing highly active catalysts with outstanding stability for industrial applications. Anisotropic nanomaterials-based electrocatalysts with optimized morphology, precise chemical composition, and tuned active sites have shown acceptable catalytic activity for the reduction of CO_2_. But when it comes to stability, there are still challenges that have to be addressed.

Recently, Bharath et al. (Bharath et al., 2022) used the surfactant-mediated synthetic approach effectively to synthesize Au nanostructures with nanorod, spherical, and nanosheet morphologies. In this study, polyethylene glycol (PEG), cetyltrimethylammonium bromide (CTAB), and n-dodecyl glyceryl itaconate (DGI) were used as surfactants to fabricate the structure. The Au nanostructures with different geometries were employed as photocatalysts for the plasmon-enhanced electrochemical reduction of N_2_ and CO_2_ to urea. This study provides strategies for the fabrication of photocathodes that can concomitantly convert N_2_ and CO_2_ into urea and are highly stable. By adjusting the applied voltage and the molar ratio of the electrolyte, the rate of yield of urea was adjusted. This study also demonstrates that N_2_ and CO_2_ co-reduction are the key factors in the synthesis of urea, whereas the roles of NO_3_^−^ and HCO_3_^−^ in the overall urea yield is minimal.

In another work, Marepally et al. demonstrated the vital role of dendritic-type morphology and associated geometrical dimensions in influencing the selectivity of Cu and Fe-based electrodes (Marepally et al., 2020). The electrodes were evaluated in a continuous flow cell at a constant potential or at the onset potential of the initial cathodic peak obtained by cyclic voltammetry. In the presence of smoother edges and when the surface sites are denser, the dendritic-type morphology has a favorable effect on the formation of formic acid, and the selectivity in the formation of gaseous and liquid products with a higher carbon chain is enhanced, as per the results obtained. The outstanding results obtained in this study demonstrate that the activity and selectivity of the electrocatalyst in the CO_2_ reduction reaction are linked to the morphology and fractal dimension more than to its chemical nature or the crystalline planes present.

**FIGURE 13:**
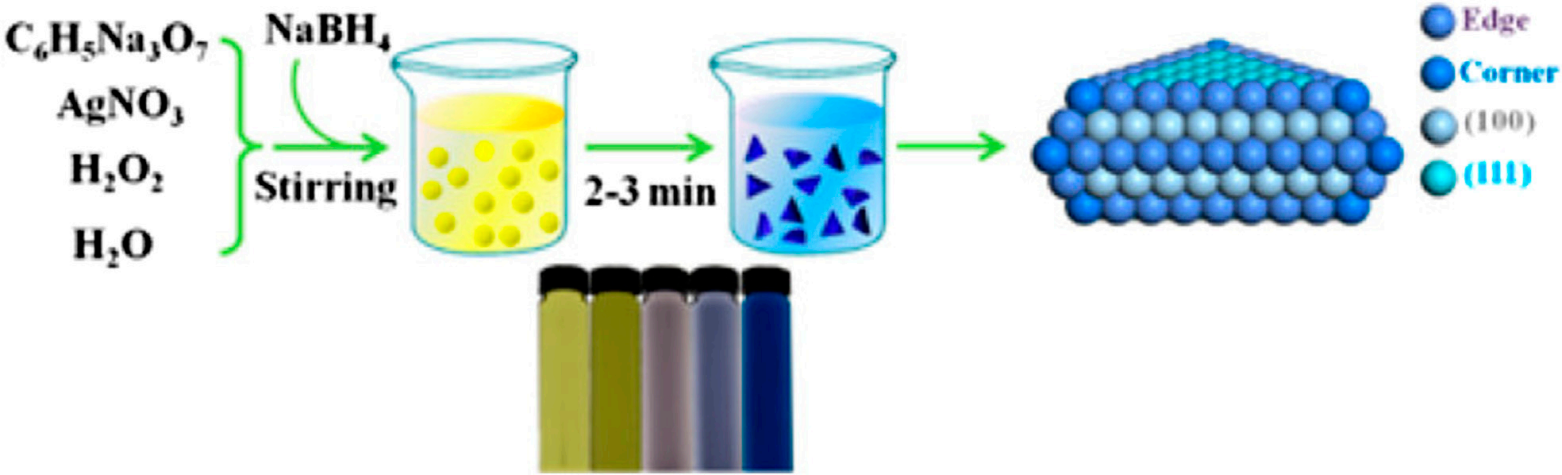
Synthesis process and digital images of Tri-Ag-NPs [Reprinted with permission from Ref. (Liu et al., 2017), American Chemical Society].

Liu et al. reported a shape-dependent preparation of CO by the electrocatalytic CO_2_ reduction using silver nanoplates with triangular shape (Tri-Ag-NPs) (Figure 13) (Liu et al., 2017). The Tri-Ag-NPs exhibited a far better current density with enhanced Faradaic efficiency as well as energy efficiency when compared with other silver nanoparticles of identical size or bulk Ag, together with a substantial stability of 7 days. Further indication that Tri-Ag-NPs are superior as catalysts for CO_2_ reduction to CO is confirmed by the observation of CO formation beginning at an ultralow overpotential of 96 mV. Calculations based on the density functional theory show that the structure, which is shape-controlled, is the reason for the much-improved electrocatalytic activity and selectivity even at lower overpotentials. Apart from the ideal edge-to-corner ratio, the Ag (100) facet dominates in the Tri-Ag-NPs with which it needs lesser energy to start the rate determining step. This research reveals a potentially useful method for tuning the activity and selectivity for the electrocatalytic CO_2_ reduction by metal catalysts via shape-controlled synthesis to produce appropriate facet and edge sites.

## 5 Conclusion and future perspectives

In this review, we attempted to provide a concise overview of the current developments in the field of anisotropic nanomaterials and their application in the field of CO_2_ utilization by catalysis. We have included an outline of the different chemical approaches that are widely employed for synthesizing anisotropic nanomaterials, as well as their key advantages and limitations. While there are many different types of materials with different and interesting morphologies reported to date, only a small number of them have been investigated for their fascinating properties in the field of catalysis and especially in the area of CO_2_ utilization. Assemblies of different anisotropic nanomaterials synthesized from different metals have been explored in light of the remarkable features of these well-organized nanostructures. Regarding the effective utilization of anisotropic nanomaterials in CO_2_ utilization, even though electrochemical and thermochemical approaches have a lot of advantages, it has been found from the literature survey that the photochemical reduction approach is the preferred and most explored one. Among the applications studied, conversion of CO_2_ to CO, synthesis of CH_4_, and methanol were the most studied ones by using the photochemical approach, while synthesis of CO, formic acid, and the concurrent conversion of N_2_ and CO_2_ to urea were the most studied ones by the electrochemical approach, and the dry reforming reaction was the one strategy employed by the thermochemical approach for the effective utilization of CO_2_. Industrial methanol production has substantial capital and maintenance expenses and is not appropriate for small-scale businesses. Because of the strong endothermic reaction in the reforming process, the procedure needs a considerable external energy source. As a result, an alternate procedure for converting methane to methanol is extremely desired. Although various procedures have been documented in academia, they are not yet practical for widespread industrial use. Furthermore, despite significant progress in the field of CO_2_ hydrogenation to ethanol, current serious issues, such as low yield and lower selectivity of ethanol obtained from both abundant transition-metal- and noble-metal-based catalysts, encourage researchers to investigate a novel strategy for ethanol synthesis by CO_2_ hydrogenation. Also, we are still not in a position to commercialize these technologies on a universal scale, which can be attributed largely to the difficulty of large-scale synthesis of these anisotropic nanomaterials as well as activity and selectivity issues pertaining to anisotropic nanomaterials. Thus, the design and development of highly active, selective, economical, environmentally benign, scalable, and stable anisotropic nanomaterials are still challenges to be undertaken by researchers around the world for a green and sustainable future.
